# Maintenance of antibody response to diphtheria/tetanus vaccine in patients aged 2–5 years with polyarticular-course juvenile idiopathic arthritis receiving subcutaneous abatacept

**DOI:** 10.1186/s12969-020-0410-x

**Published:** 2020-02-22

**Authors:** Hermine I. Brunner, Nikolay Tzaribachev, Gabriel Vega Cornejo, Rik Joos, Elisabeth Gervais, Rolando Cimaz, Inmaculada Calvo Penadés, Rubén Cuttica, Thomas Lutz, Pierre Quartier, Yash Gandhi, Marleen Nys, Robert Wong, Alberto Martini, Daniel J. Lovell, Nicolino Ruperto, for the Pediatric Rheumatology Collaborative Study Group and the Paediatric Rheumatology International Trials Organisation, Jordi Anton, Graciela Espada, Bernard Lauwerys, Ingrid Louw, Kirsten Minden, Mahmood Moosa Ally, Riana Van Zyl, Diego Oscar Viola, Richard Mouy, Brigitte Bader-Meunier, Michaela Semeraro

**Affiliations:** 1 Cincinnati Children’s Hospital Medical Center, Cincinnati, OH USA; 2 Pediatric Rheumatology Research Institute, Bad Bramstedt, Germany; 3 CREA de Guadalajara, Guadalajara, Jalisco Mexico; 4 Universitair Ziekenhuis Ghent, Ghent, Belgium; 5 CHU de Poitiers, Rheumatology, Poitiers, France; 6 Department of Clinical Sciences and Community Health, University of Milano, Milan, Italy; 7 Azienda Ospedaliera Universitaria Anna Meyer, Florence, Italy; 8 Hospital Universitario y Politécnico de La Fe, Valencia, Spain; 9 Hospital General de Niños Pedro de Elizalde, Buenos Aires, Argentina; 10 Center for Pediatric and Adolescent Medicine/Pediatric Rheumatology, University Hospital Heidelberg, Heidelberg, Germany; 11 Paris University, IMAGINE Institute, RAISE reference centre, Necker-Enfants Malades Hospital, Assistance Publique-Hopitaux de Paris, Paris, France; 12 Bristol-Myers Squibb, Princeton, NJ USA; 13 Bristol-Myers Squibb, Braine-L’Alleud, Belgium; 14 Istituto Giannina Gaslini, Genoa, Italy

**Keywords:** Juvenile idiopathic arthritis, Abatacept, Biologic DMARDs, Vaccination

## Abstract

**Background:**

Patients with polyarticular-course juvenile idiopathic arthritis (pJIA), receiving disease-modifying anti-rheumatic drugs with immunosuppressive effects, may be at increased risk of vaccine-preventable infections. This substudy assessed protective antibody responses to diphtheria and tetanus vaccination given prior to study enrolment in patients with pJIA.

**Findings:**

This was a substudy of a 24-month, single-arm, open-label, multicenter, Phase III trial (NCT01844518) of subcutaneous abatacept in children with active pJIA (*N* = 219). Patients aged 2–5 years, with ≥2 continuous months of weekly weight-tiered (10–< 25 kg [50 mg], 25–< 50 kg [87.5 mg]) subcutaneous abatacept treatment (with/without methotrexate and/or low-dose corticosteroids), who received diphtheria/tetanus vaccine prior to enrolment, were eligible. Protective antibody levels to diphtheria/tetanus (> 0.1 IU/mL), and safety, were assessed.

Overall, 29 patients were analyzed: 19 (65.5%), 1 (3.4%) and 9 (31.0%) patients had > 12, 6–12 and 2–< 6 months of abatacept exposure, respectively. All patients had protective antibody levels to tetanus and 26 (89.7%) patients had protective antibody levels to diphtheria. Of the 3 patients without protective antibody levels to diphtheria, each had an antibody level of 0.1 IU/mL, bordering the lower threshold of protection. Concomitant use of methotrexate and/or low-dose corticosteroids had no evident effect on antibody levels. No unexpected adverse events, including cases of diphtheria or tetanus, were reported during the 24-month period.

**Conclusions:**

Patients aged 2–5 years with pJIA who received 2–24 months of weekly subcutaneous abatacept, with or without concomitant methotrexate and/or low-dose corticosteroids, maintained effective diphtheria and tetanus vaccination protection without new safety signals.

**Trial registration:**

ClinicalTrials.gov (NCT01844518); registered May 1, 2013; https://clinicaltrials.gov/ct2/show/NCT01844518?term=NCT01844518&rank=1

## Findings

### Introduction

Patients with polyarticular-course juvenile idiopathic arthritis (pJIA) receiving disease-modifying antirheumatic drugs (DMARDs) with immunosuppressive effects may be at increased risk of vaccine-preventable infections such as diphtheria and tetanus. The European League Against Rheumatism recommendations for the vaccination of children with rheumatic diseases endorse adherence to national guidelines for very young, healthy children [1]. However, due to ongoing maturation of the immune system in young children [2, 3], immune responses to vaccination in this population may be compromised by immunosuppressive medications more so than in older children [4].

Abatacept, a selective T-cell co-stimulation modulator [5], was effective and well-tolerated in children with pJIA aged 2–17 years (subcutaneous [SC] formulation) [6], and in children aged 6–17 years (intravenous [IV] formulation) [7]. In healthy adults, a single 750-mg dose of IV abatacept did not prevent induction of protective antibodies to tetanus toxoid or standard 23-valent pneumococcal vaccine [8]. Adults with rheumatoid arthritis (RA) who received ≥3 months’ SC abatacept treatment (125 mg/week with concomitant methotrexate [MTX] and/or low-dose corticosteroids) could mount an appropriate immune response to the 23-valent pneumococcal vaccine and 2011–2012 trivalent seasonal influenza vaccine [9]. However, studies investigating the effects of abatacept on vaccination in very young children with JIA are lacking. This substudy assessed protective antibody response to diphtheria and tetanus vaccination given prior to study enrolment in patients aged 2–5 years with pJIA.

## Methods

### Study design and patient population

This substudy of a 24-month, Phase III trial (NCT01844518) of SC abatacept in patients (cohort 1: 173 patients aged 6–17 years; cohort 2: 46 patients aged 2–5 years) with active pJIA and inadequate response/intolerance to ≥1 DMARD [6] was conducted across 48 centers worldwide by members of the Paediatric Rheumatology International Trials Organisation [10] and the Pediatric Rheumatology Collaborative Study Group [11]. Patients received weight-tiered weekly SC abatacept (10–< 25 kg [50 mg], 25–< 50 kg [87.5 mg]) for 4 months. JIA-American College of Rheumatology criteria 30% improvement responders at Month 4 could receive treatment for another 20 months. Stable doses of concomitant MTX (≤30 mg/m^2^/week) and low-dose oral corticosteroids (≤10 mg/day or ≤ 0.2 mg/kg/day [whichever was less] prednisone equivalent) were permitted if used at baseline. The concomitant use of a tumour necrosis factor inhibitor (TNFi) was prohibited; 10/46 (22%) patients had prior TNFi treatment (adalimumab, etanercept or tocilizumab) [6]. Patients from cohort 2, with ≥2 continuous months of abatacept treatment, who received diphtheria/tetanus vaccine prior to enrolment and who were consented to participate by their legal guardians, were included in this substudy. A 60-day washout period was required for other biologics prior to the first dose of abatacept; therefore, levels of other biologics would be non-existent and should not influence the results of this analysis.

### Outcomes and analysis

A single blood sample was obtained to assess antibody levels to tetanus and diphtheria. Assessment of blood antibody levels was performed by a central laboratory (ARUP Laboratories, Salt Lake City, UT, USA) using quantitative multiplex bead assays [12]. Protective antibody levels to diphtheria and tetanus were defined as > 0.1 IU/mL by the central laboratory, Centers for Disease Control and Prevention (CDC) and World Health Organization (WHO) criteria [12–15]. Immunogenicity of the pertussis component of this vaccine was not studied, as there is no established WHO-defined measure of protection against pertussis following immunization or natural infection [16] and the central laboratory used WHO-defined protective antibody levels for diphtheria and tetanus [13, 14]. Potential impact of concomitant immunosuppressive medications (MTX and/or low-dose corticosteroids [prednisone]) on diphtheria and tetanus antibody levels was evaluated descriptively by medication at the time of blood sample collection. Patient demographics, antibody level data and safety were analyzed descriptively.

## Results

### Patient disposition and baseline characteristics

Overall, 29/46 (63.0%) patients from cohort 2 participated in this substudy. Baseline characteristics of the patients who participated versus those who did not participate were similar (Table 1). Among participants, 19 (65.5%), 1 (3.4%) and 9 (31.0%) had > 12, 6–12 and 2–< 6 months of abatacept exposure, respectively. All patients were vaccinated before abatacept initiation; one patient received a single vaccination during the study after 3 months of abatacept treatment.

**Table 1:** Baseline demographics and disease characteristics of patients who participated in the vaccination substudy and of those who did not

	Participated *n* = 29	Did not participate *n* = 17	Total cohort 2^a^ *n* = 46
Age at enrolment, years	4.2 (0.9)	3.8 (1.2)	4.1 (1.0)
Female, *n* (%)	16 (55.2)	12 (70.6)	28 (60.9)
Weight, kg	18.4 (3.6)	18.0 (3.5)	18.2 (3.5)
Weight categories, kg, *n* (%)			
< 25	27 (93.1)	16 (94.1)	43 (93.5)
25–50	2 (6.9)	1 (5.9)	3 (6.5)
≥ 50	0	0	0
Race, *n* (%)			
White	29 (100.0)	15 (88.2)	44 (95.7)
Black/African American	0	1 (5.9)	1 (2.2)
Other	0	1 (5.9)	1 (2.2)
Geographic region, *n* (%)			
South America	4 (13.8)	4 (23.5)	8 (17.4)
Europe	25 (86.2)	11 (64.7)	36 (78.3)
Rest of world	0	2 (11.8)	2 (4.3)
Duration of JIA, years	0.8 (1.0)	0.8 (1.0)	0.8 (1.0)
JIA disease onset categories, *n* (%)			
Polyarthritis RF−	18 (62.1)	12 (70.6)	29 (63.0)
Polyarthritis RF+	0	2 (11.8)	3 (6.5)
Extended oligoarthritis	8 (27.6)	2 (11.8)	10 (21.7)
Psoriatic arthritis	3 (10.3)	1 (5.9)	4 (8.7)
No. of active joints	10.0 (6.4)	8.1 (5.2)	9.3 (6.0)
No. of joints with limitation of motion	9.6 (5.9)	7.1 (4.8)	8.7 (5.6)
CHAQ-DI score	1.0 (0.7)	1.2 (0.6)	1.1 (0.7)
Parent global assessment score	37.0 (22.9)	41.4 (26.0)	38.6 (23.9)
Physicians global assessment score	50.2 (16.1)	41.9 (20.4)	47.1 (18.0)
CRP, mg/dL	0.8 (1.4)	2.3 (3.4)	1.3 (2.4)
MTX dose ay Day 1, mg/m^2^/week	*n* = 22 13.5 (4.5)	*n* = 14 12.0 (2.3)	*n* = 37 12.9 (3.8)
Prednisone dose at Day 1, mg/kg/day	*n* = 3 0.2 (0.0)	*n* = 5 0.3 (0.2)	*n* = 8 0.2 (0.1)

Data are mean (SD) unless otherwise specified. Baseline is Day 1 of the study or the last measurement prior to short-term dose. Cohort 2 included all patients aged 2–5 years. Minor differences between the data for total cohort 2 and those for the participated patients and patients who did not participate in the substudy are due to the different dates of database lock (participated patients and patients who did not participate: July 2, 2018; total cohort 2: February 16, 2017)

^a^Includes all patients who did or did not participate in the vaccination substudy

*CHAQ-DI* Childhood Health Assessment Questionnaire-Disability Index, *CRP* C-reactive protein, *JIA* juvenile idiopathic arthritis, *MTX* methotrexate, *RF* rheumatoid factor

### Protective antibody assessment

Antibody assessment in individual patients is presented in Table 2. All patients had protective antibody levels to tetanus after ≥2 months of abatacept treatment and 26/29 (89.7%) patients had protective antibody levels to diphtheria. Of the remaining 3 patients (Table 2; patients 18, 20 and 24), each had a protective antibody level to diphtheria of 0.1 IU/mL, which bordered the lower threshold of protection [12, 14]. These 3 patients received 4 injections (3 initial injections and one booster shot) of combined diphtheria, hepatitis B, *Haemophilus influenzae* type b, pertussis, poliomyelitis and tetanus vaccine or combined diphtheria, tetanus and pertussis vaccine with 21–49 months between last injection and abatacept initiation and 24–79 months between last injection and antibody assessment. No differences were noted in types of vaccines received by, or in the vaccine schedules of, patients who maintained protective antibody levels to diphtheria or the 3 patients with borderline levels. Concomitant use of MTX and/or low-dose corticosteroids had no evident effect on antibody levels: 19/20 (95.0%) patients receiving MTX and/or low-dose corticosteroids maintained protective levels to diphtheria and tetanus compared with 7/9 (77.8%) patients receiving no MTX or corticosteroids.

**Table 2:** Line listing of baseline characteristics, treatment and antibody assessment of patients

Patient number	Age, sex (years; female or male)	Abatacept dose (mg); duration of exposure^a^ (months)	Number of vaccine injections	Duration between last vaccination and abatacept initiation (months)	Duration between last vaccination and antibody sample collection (months)	Blood antibody level to diphtheria^b^ (IU/mL)	Blood antibody level to tetanus^b^ (IU/mL)	Concomitant MTX dose^c^ (mg/m^2^/week; oral or SC)	Concomitant prednisone dose^c^ (mg/kg/day)
1	5, F	50; 27	N/A	N/A	N/A	0.6	5.4	–	–
2	3, F	50; 19	4	30	49	0.8	0.4	7.9, O	–
3	3, M	50; 4	4	27	30	0.2	0.8	16.6, O	–
4	5, F	50; 24	4	−3	21^d^	0.8	2.2	15.5, O	–
5	5, F	50; 22	5	N/A	N/A	0.5	4.9	3.4, O	–
6	5, F	50; 22	4	52	73	0.9	1.0	9.9, O	–
7	4, M	87.5^e^; 19	4	47	66	0.5	5.9	–	–
8	4, M	50; 19	5	43	62	0.7	8.2	13.1, SC	–
9	4, F	50; 16	3	52	67	0.5	0.6	16.2, SC^f^	–
10	5, M	50; 19	5	54	72	0.7	8.3	16.0, SC	–
11	3, F	50; 19	4	36	54	0.5	0.4	–	–
12	4, M	87.5; 4	4	35	39	0.4	2.3	14.0, O	–
13	3, F	50; 4	4	30	34	0.6	2.0	11.7, SC	–
14	4, F	50; 2	4	46	48	0.5	1.8	10.4, SC	–
15	2, F	50; 2	3	24	26	0.6	3.1	14.3, SC	–
16	5, M	50; 3	4	57	60	0.4	0.6	–	–
17	5, M	87.5; 3	4	57	60	0.3	1.1	–	–
18	2, F	50; 3	4	21	24	0.1	0.2	–	–
19	4, F	50; 3	4	46	49	0.5	1.4	11.5, SC	–
20	5, M	50; 30	4	49	79	0.1	0.3	–	–
21	5, M	50; 24	5	54	78	1.1	5.7	10.0, SC	–
22	4, F	87.5^e^; 24	4	45	69	1.5	23.2	–	–
23	5, M	50; 16	4	31	47	0.2	3.2	–	–
24	4, M	87.5^e^; 16	4	35	51	0.1	1.8	11.7, O	–
25	5, F	50; 19	N/A	N/A	N/A	0.5	0.8	22.9, O	0.1
26	5, F	87.5^e^; 19	N/A	N/A	N/A	3.8	3.8	11.5, O	–
27	5, M	50; 19	N/A	N/A	N/A	0.6	3.3	20.1, O	0.1
28	4, M	87.5^g^; 21	N/A	N/A	N/A	0.5	0.5	14.8, O	–
29	5, F	87.5^e^; 10	N/A	N/A	N/A	8.0	12.5	12.2, O	–

^a^At time of blood sample collection

^b^Protective antibody threshold > 0.1 IU/mL

^c^Concurrent medication use assessed at the time of blood sample collection, or at the closest date before with available status

^d^A single vaccination was administered during the study period after 3 months of abatacept treatment. Antibody sample collection was carried out 21 months after the final vaccination dose was administered

^e^Patient initiated abatacept 50 mg before dose escalation

^f^Patient switched from 16.2 mg/m^2^/week SC MTX to 12.9 mg/m^2^/week oral MTX on the day of blood sample collection

^g^Patient initiated abatacept 50 mg, received 5 doses of abatacept at 125 mg before returning to 50 mg, and later escalated to 87.5 mg

*F* female, *M* male, *MTX* methotrexate, *N/A* not available, *O* oral, *SC* subcutaneous

### Safety

A safety summary of cohort 2 is presented in Table 3. Abatacept safety profile was consistent between age cohorts [6]. Related serious adverse events (SAEs), SAEs and related AEs were reported in a higher proportion of patients who participated versus those who did not participate in this substudy. Due to the relatively small sample size, these data should be interpreted with caution. No cases of diphtheria or tetanus, or symptoms suggestive of an untoward reaction to the vaccine, were reported during the 24-month period.

**Table 3:** Safety summary for patients who participated in the vaccination substudy and for those who did not

Event	Participated *n* = 29	Did not participate *n* = 17	Total cohort 2^a^ *n* = 46
Deaths	0	0	0
SAEs	4 (13.8)^b^	1 (5.9)	5 (10.9)
Related SAEs^c^	2 (6.9)	0	2 (4.3)
Discontinued due to SAEs	0	0	0
AEs	29 (100.0)	15 (88.2)	44 (95.7)
Related AEs	23 (79.3)	7 (41.2)	30 (65.2)
Discontinued due to AEs	0	1 (5.9)	1 (2.2)

Data are number (%) of patients. Cohort 2 included all patients aged 2–5 years

^a^Includes all patients who did or did not participate in the vaccination substudy

^b^SAEs included febrile convulsions, tendon disorder, limb abscess, cellulitis and an overdose of mild intensity (administration of a higher abatacept dose due to misclassification by weight tier)

^c^Treatment-related SAEs included a limb abscess that was severe in intensity and an overdose of mild intensity (administration of a higher abatacept dose due to misclassification by weight tier)

*AE* adverse event, *SAE* serious adverse event

## Discussion

In this substudy of patients aged 2–5 years with pJIA and prolonged exposure to SC abatacept, all patients maintained protective antibody levels to tetanus, and all but 3 to diphtheria following vaccination prior to study enrolment. Addition of MTX and/or low-dose corticosteroid to SC abatacept treatment did not appear to prevent the maintenance of protective antibody levels in this population. Immune system maturation takes place over the early years of life [2, 3]; therefore, ensuring that very young patients who are receiving immunosuppressive medication can maintain protective antibody levels in response to vaccination is important.

According to the CDC, a complete vaccine series leads to development of protective antibody levels in nearly 100% of healthy children for tetanus and 95% for diphtheria [15], which corresponds to the findings of this study. In the substudies of two trials that included adults with RA who received ≥3 months of treatment with abatacept, 74% of patients achieved an immunological response to influenza vaccination and 61% to standard 23-valent pneumococcal polysaccharide vaccine [9], similar to the responses seen in the general population [17, 18]. Importantly, in the present trial, patients were vaccinated before abatacept treatment, whereas in the aforementioned trials, vaccination was administered to patients after treatment with abatacept.

Published research of vaccination in patients with JIA receiving treatment with biologics is limited. Among 15 patients with JIA aged 6–17 years, neither low-dose MTX nor etanercept caused statistically relevant differences in protective antibody levels following measles, mumps and rubella vaccination compared with untreated healthy controls [19]. Similarly, among 27 patients with mean (standard deviation [SD]) age of 10.4 (5.6) years with systemic-onset JIA who received tocilizumab for a mean (SD) of 1.9 (1.4) years and 17 healthy controls, efficacy of influenza vaccination did not differ significantly between the groups [20]. In addition, in a double-blind, randomized controlled trial, anakinra treatment did not prevent the generation or maintenance of protective antibody levels to standard 23-valent pneumococcal vaccine after 12 months in patients with systemic-onset JIA and a mean (SD) age of 9.5 (5.2) years [21]. In a study of the effects of TNFi treatment on the immunogenicity of 7-valent conjugate pneumococcal vaccine in patients with JIA aged 4–18 years, 87–100% of patients generated protective antibody levels, depending on vaccine serotype [22].

As shown in other studies evaluating vaccination in patients with JIA (reviewed in Groot N, et al. [4]), including a large retrospective cross-sectional analysis [23], concomitant treatment with MTX and/or low-dose corticosteroids does not prevent the generation of protective antibody titres in patients with JIA. Patients receiving treatment with corticosteroids may show lower seroconversion rates, but they usually still reach protective antibody titres [24].

The limitations of this study should be considered. All patients included in this study were of a similar background (100% white; 86% from Europe), which may limit the generalizability of the findings. In addition, the sample size was relatively small and as the analysis was not a classically designed vaccination study, where vaccination is administered during the trial, it was not possible to determine if the patients with borderline protective antibody titres ever had protective levels following vaccination, or the rate at which these titres decreased; therefore, the data should be interpreted with caution.

Overall, paediatric patients with pJIA as young as 2 years old who received 2–24 months of SC abatacept treatment, with the possible addition of MTX and low-dose corticosteroids, were able to maintain effective diphtheria and tetanus vaccination protection without unexpected AEs. These results show that SC abatacept does not prevent the maintenance of protective antibody levels against tetanus and diphtheria, even if the booster dose was not administered recently.

## Data Availability

Bristol-Myers Squibb policy on data sharing may be found at https://www.bms.com/researchers-and-partners/clinical-trials-and-research/disclosure-commitment.html

## Abbreviations

AE: Adverse event
CDC: Centers for Disease Control and Prevention
DMARD: Disease-modifying antirheumatic drug
IV: Intravenous
JIA: Juvenile idiopathic arthritis
MTX: Methotrexate
pJIA: Polyarticular-course juvenile idiopathic arthritis
RA: Rheumatoid arthritis
SAE: Serious adverse event
SC: Subcutaneous
SD: Standard deviation
TNFi: Tumour necrosis factor inhibitor
WHO: World Health Organization
